# Fear and depression during the COVID-19 outbreak in Cameroon: a nation-wide observational study

**DOI:** 10.1186/s12888-021-03323-x

**Published:** 2021-07-15

**Authors:** Joseph Nelson Siewe Fodjo, Leonard Ngarka, Wepnyu Y. Njamnshi, Leonard N. Nfor, Michel K. Mengnjo, Edwige Laure Mendo, Samuel A. Angwafor, Jonas Guy Atchou Basseguin, Cyrille Nkouonlack, Edith N. Njit, Nene Ahidjo, Eric S. Chokote, Fidèle Dema, Julius Y. Fonsah, Godwin Y. Tatah, Nancy Palmer, Paul F. Seke Etet, Dennis Palmer, Dickson S. Nsagha, Daniel E. Etya’ale, Stephen Perrig, Roman Sztajzel, Jean-Marie Annoni, Anne-Cécile Zoung-Kanyi Bissek, Rose G. F. Leke, Marie-Thérèse Abena Ondoa Obama, John N. Nkengasong, Robert Colebunders, Alfred K. Njamnshi

**Affiliations:** 1 Global Health Institute, University of Antwerp, Antwerp, Belgium; 2 Brain Research Africa Initiative (BRAIN), Yaoundé, Cameroon; 3 Brain Research Africa Initiative (BRAIN), Geneva, Switzerland; 4 Division of Health Operations Research, Ministry of Public Health, Yaoundé, Cameroon; 5 CDC Africa, African Union, Addis Ababa, Ethiopia

**Keywords:** COVID-19, PHQ-9, FCV-19S, Cameroon, Fear, Depression

## Abstract

**Background:**

The COVID-19 pandemic has been associated with significant psychological and social distress worldwide. We investigated fear and depression among adults in Cameroon during different phases of the COVID-19 outbreak.

**Methods:**

An online survey was conducted in Cameroon from June–December 2020 using a structured questionnaire. Socio-demographic data and information regarding COVID-19 history were obtained. Fear and depressive symptoms were assessed using the Fear of COVID-19 score (FCV-19S) and the Patient Health Questionnaire (PHQ-9), respectively. Responses were clustered in weeks to better appreciate their evolution over time.

**Results:**

Overall, 7381 responses from all ten regions of Cameroon were analysed (median age: 30 years, 73.3% male). The prevalence of depression (PHQ-9 score ≥ 10) was 8.4%, and that of high fear of COVID-19 (FCV-19S scores ≥19) was 57.4%. These rates were similar across genders, age-groups, and region of residence. While mean weekly PHQ-9 scores remained fairly stable throughout the study period (range: 2.53–3.21; *p* = 0.101), mean FCV-19S scores were highest during the early weeks but decreased significantly thereafter (from 20.31 to 18.34; *p* <  0.001). Multivariate analyses revealed that having a postgraduate degree, a history of quarantine, flu-like symptoms during the past 14 days, and higher FCV-19S scores were associated with more severe depressive symptoms, while obtaining COVID-19 information from various sources reduced the odds for depression.

**Conclusion:**

Depression amidst the COVID-19 crisis is less prevalent in Cameroon than in other countries. Prompt and widespread dissemination of adequate COVID-19 information may reduce the risks for depression by dispelling fear and anxiety among Cameroonians.

## Background

Since its initial outbreak in China in December 2019, the coronavirus disease 2019 (COVID-19) has spread globally and caused over a million deaths [1]. Unprecedented restrictive measures were deployed by every nation to prevent or contain local epidemics. Besides the biological (and sometimes fatal) damages caused by the virus, the COVID-19 pandemic has been associated with significant psychological and social distress around the globe. Indeed, studies conducted during the COVID-19 pandemic have revealed high levels of stress, anxiety and depressive disorders among the general population [2], as well as specific populations: healthcare workers and their families [3–5], military personnel [6], and COVID-19 survivors [7]. Available data suggests that women and younger individuals are more affected by psychosocial symptoms during the COVID-19 pandemic [8, 9]. The socio-economic consequences of the strict lockdown measures implemented in many countries further exacerbated the financial insecurities of the population with adverse effects on their psychological and social well-being, leading to suicide in some cases [10].

Cameroon, a Low-Middle income country located in the Central African sub-region, was not spared by the COVID-19 pandemic. After reporting its first case on March 6th 2020, community transmission was observed resulting in a cumulative 26,277 cases and 448 deaths as of December 31st 2020 [11]. The necessary preventive measures prescribed by the Cameroonian government [12] have disrupted daily routines and adversely impacted the nation’s socio-economic landscape given that a large proportion of the population relies on the informal sector for its livelihood. Although the mental or brain health impacts of the COVID-19 pandemic are evident in Cameroon [13], the magnitude of the problem is still poorly understood as no nation-wide study has yet been done. We therefore conducted this study to assess the level of fear and the frequency of depressive disorders in the Cameroonian population amidst the COVID-19 outbreak. We further sought to identify determinants of fear and depressive symptoms during the ongoing health crisis.

## Methods

### Study setting and population

The study was conducted from June 5th to December 5th 2020, and recruited participants from all ten regions of Cameroon. Cameroon’s population has a median age of 18.7 years and life expectancy of 60.3 years, with 56.3% of people living in urban settings [14]. For over 4 years, the nation has been confronted with an internal conflict in the two English-speaking regions (North West and South West) and sporadic terrorist attacks in the Northern part of the country.

### Study tools and procedures

We performed online surveys designed by Brain Research Africa Initiative (BRAIN) researchers in collaboration with the International Citizen Project on COVID-19 (ICPCovid) consortium [15]. The ICPCovid website, initiated by a team of researchers based at the University of Antwerp in Belgium, offers a secure electronic platform to collect COVID-19-related data from several low- and middle-income countries in order to assess the population’s perception of, and adherence to the implemented preventive measures. A web-based online questionnaire was designed using the ‘Drupal’ system, translated to English and French and pre-tested on Cameroonian adults. Besides collecting the socio-demographic information of participants, we also assessed their psychological and social well-being using two scales:

- Fear of COVID-19 scale (FCV-19S): This is a 7-item tool that was recently validated in Iran [16]. Each item on this scale is scored between 1 and 5. A total score is calculated by adding up each item score (overall score ranging from 7 to 35). The higher the score, the greater the fear of cororonavirus-19. A cut-off score of 19 and above on this scale has been suggested the identify individuals with a high level of COVID-19-related fear [17].
- Patient Health Questionnaire-9 (PHQ-9): This 9-item tool was developed to screen for major depressive disorders among adults [18]. Each of the nine items is scored between 0 and 3 (maximum possible score: 27). Scores of 10 or above are often considered as an indication of a likely depressive disorder, as previously validated in a Cameroonian study population [19]. Other cut-offs have been proposed to further discriminate the severity of depressive symptoms: cut-off scores of 0–4 = none/minimal; 5–9 = mild; 10–14 = moderate; 15–19 = moderately severe; 20–27 = severe [3, 17].

In addition to the above-mentioned screening tools, Likert-format questions were asked regarding the level of worry experienced by the respondents regarding their own health and the health of their loved ones during the COVID-19 crisis; this was expressed on a 5-point scale ranging from 1 (not at all worried) to 5 (extremely worried). The web-link to the electronic survey was disseminated via social media platforms and bulk messaging to phone users. Upon clicking on the link, the user was directed to an information and consent page where he/she could agree to participate, fill in the responses submit them via a smartphone, tablet, or computer. The electronic questionnaire was made accessible during certain periods each month, and closed down intermittently between survey rounds. All submitted responses were immediately stored in a password-protected server in Belgium until data retrieval.

### Data analysis

Collected data were exported to Microsoft Excel 2016 spreadsheets for cleaning, and later transferred to R version 4.0.2 for analysis. Based on the fact that continuous variables were not normally distributed as shown by the Kolgomorov-Smirnov test, we summarized them as median with interquartile range (IQR). On the other hand, categorical variables were expressed as fractions and percentages. To investigate determinants of depressive symptoms as screened by the PHQ-9 tool, an ordinal logistic regression model was constructed with the different severity levels of depression (none/minimal, mild, moderate, moderately severe, severe) as dependent variable and socio-demographic variables as covariates. To capture the effect of the ongoing armed conflicts in some parts of the country, a binary variable was created as follows: conflict-stricken region = yes (for respondents residing in the Far North, North West, and South West regions) vs no (for all other regions). Given the prolonged study period and the rapid evolution of COVID-19 dynamics, we took into account the timing of the responses by considering the different study weeks as clusters. Only weeks with ≥100 responses were included in the multivariate analysis. We used the *polr* function (package: ‘MASS’) and the *vcovCL* function (package: ‘sandwich’) in the software R to obtain clustered standard errors from the regression model. Covariates for the final model were selected based on a *p*-value< 0.2 during univariate analysis.

## Results

Of the 7538 responses received, 7381 were eligible for analysis (median age: 30 years, 73.3% male); Table 1. About one-fifth of the participants (*n* = 1454; 19.7%) resided in the conflict-stricken regions of Cameroon. Majority of participants (70.9%) had attained university level of education.

**Table 1:** Participants’ characteristics

Characteristics	Survey findings ***N*** = 7381
Age: Median [IQR]	30.0 (25.0–38.0)
Gender: n (%)	
Male	5409 (73.3%)
Female	1972 (26.7%)
Highest educational level: n (%)	
Primary school	118 (1.6%)
Secondary school	2029 (27.5%)
University: Undergraduate	2963 (40.1%)
University: Postgraduate	2271 (30.8%)
Residential setting: n (%)	
Rural	672 (9.1%)
Sub-Urban	1327 (18.0%)
Urban	5382 (72.9%)
Living alone in household: n (%)	1383 (18.7%)
Self-reported socio-economic status: n (%)	
Low class	2494 (33.8%)
Lower-middle class	3839 (52.0%)
Upper-middle class	912 (12.4%)
High class	136 (1.8%)
Profession: n (%)	
Student	1832 (24.8%)
Unemployed	1342 (18.2%)
Self-employed	869 (11.8%)
Private employee	1843 (25.0%)
Government employee	1285 (17.4%)
Retired	210 (2.9%)
Healthcare worker or student: n (%)	793 (10.7%)
Source of COVID-19 information: n (%)^a^	
Radio, Television, or government announcements	6601 (89.4%)
Social Media	5780 (78.3%)
Healthcare worker	2874 (38.9%)
Underlying chronic disease: n (%)^b^	716 (9.7%)
History of being quarantined at home/institution: n (%)	1375 (18.6%)
History of violence/discrimination during COVID-19 outbreak: n (%)	1334 (18.1%)

*IQR* Interquartile range [25th percentile – 75th percentile]

^a^Each participant was allowed to choose more than one answer, hence the categories may overlap

^b^Heart disease, diabetes, hypertension, cancer, HIV, or asthma

The median PHQ-9 score in our study population was 1.0 (IQR: 0–4) on a scale ranging from 0 to 21. Using the PHQ-9 cut-off value of ≥10 as positive screening for depression, the prevalence of depression was 617/7381 (8.4%). Applying the other cut-offs revealed the frequencies of no/minimal, mild, moderate, moderately severe, and severe depression to be respectively: 5669 (76.8%), 1095 (14.8%), 346 (4.7%), 178 (2.4%), and 93 (1.3%). Regarding COVID-19 fear, FCV-19S scores ranged from 7 to 35, with median 20 (IQR: 15–23) and mean 19.3 ± 6.4. Furthermore, more than half of the respondents (*n* = 4238; 57.4%) reported experiencing high levels of fear (i.e. FCV-19S scores ≥19). The prevalence of both depression (PHQ-9 score ≥ 10) and high level of fear (FCV-19S ≥19) were similar across genders, age groups, residential setting, being in the healthcare sector or not, and region of residence (Table 2).

**Table 2:** Prevalence of depression and high fear levels during the COVID-19 outbreak in Cameroon

Characteristics		FCV-19S ≥ 19: n (%)	***P***-value^a^	PHQ-9 score ≥ 10: n (%)	***P***-value^a^
Gender	Male	3079 (56.9%)	0.163	458 (8.5%)	0.611
	Female	1159 (58.8%)		159 (8.1%)	
Age	18–25 years	1186 (56.7%)	0.289	185 (8.8%)	0.588
	26–35 years	1724 (57.4%)		254 (8.5%)	
	36–45 years	750 (56.4%)		104 (7.8%)	
	46–55 years	365 (60.9%)		51 (8.5%)	
	> 55 years	213 (60.0%)		23 (6.5%)	
Region	Centre	1267 (57.8%)	0.758	178 (8.1%)	0.773
	Adamawa	183 (57.4%)		29 (9.1%)	
	East	101 (55.8%)		9 (5.0%)	
	Far North	247 (58.1%)		43 (10.1%)	
	Littoral	1006 (57.0%)		147 (8.3%)	
	North	131 (55.3%)		19 (8.0%)	
	North West	414 (56.6%)		67 (9.2%)	
	South	129 (54.9%)		18 (7.7%)	
	South West	440 (60.9%)		59 (8.2%)	
	West	320 (55.8%)		48 (8.4%)	
Residential setting	Rural	374 (55.7%)	0.624	51 (7.6%)	0.646
	Sub-urban	765 (57.6%)		107 (8.1%)	
	Urban	3099 (57.6%)		459 (8.5%)	
Worker/student in healthcare	Yes	432 (54.5%)	0.083	67 (8.5%)	0.977
	No	3806 (57.8%)		550 (8.4%)	

^a^Chi-Squared test

**Fig. 1:**
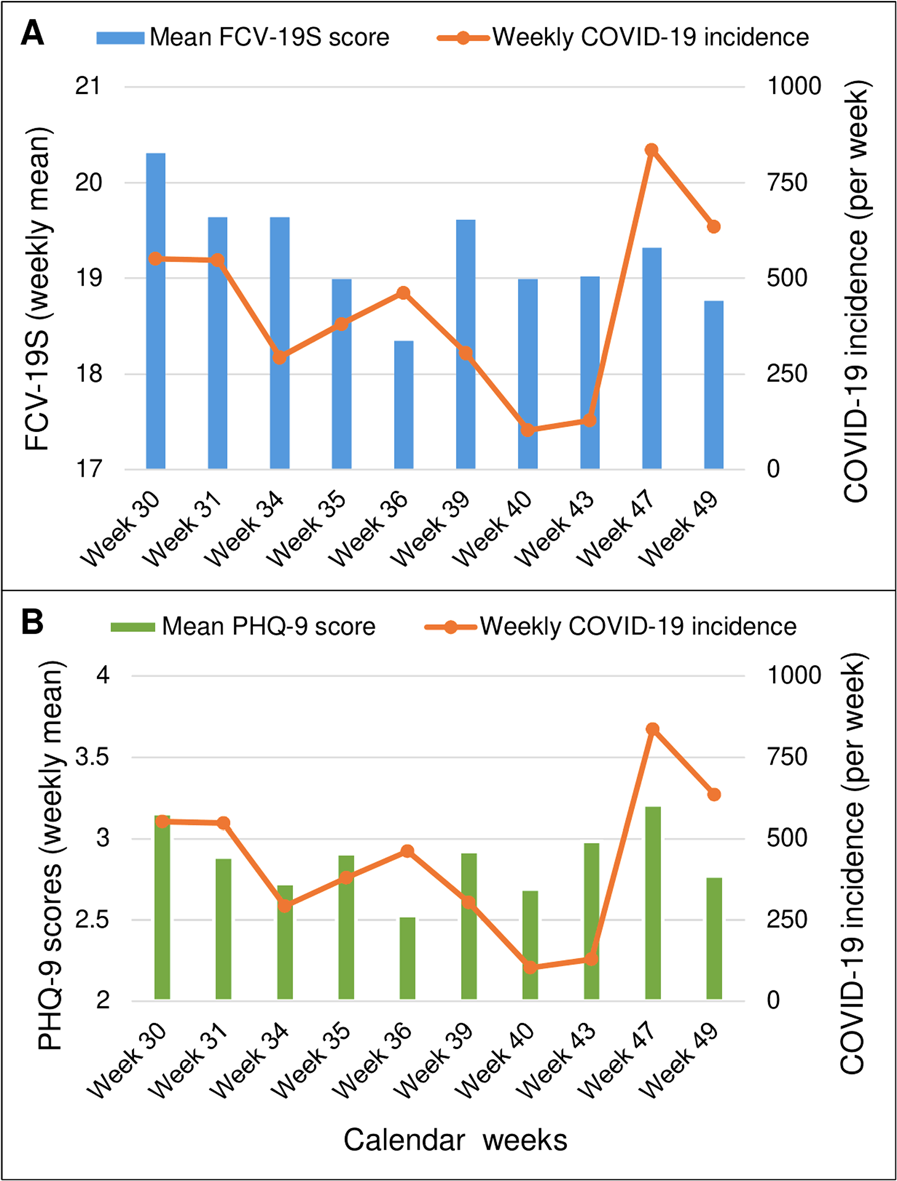
Weekly evolution of COVID-19 incidence and: **A** Fear scores; **B** PHQ-9 scores

FCV-19S scores correlated significantly with PHQ-9 scores (Spearman-rho = 0.28, *p* <  0.001). Considering only weeks with at least 100 responses, both COVID-19 fear and PHQ-9 scores seemed to increase with increasing weekly incidence of COVID-19 in Cameroon, and vice versa (Fig. 1). While the COVID-19 weekly incidence in Cameroon did not correlate significantly with the respondents’ FCV-19S scores (Spearman-rho = 0.021, *p* = 0.078), a weak positive correlation was found between weekly COVID-19 incidence and PHQ-9 scores (Spearman-rho = 0.033, *p* = 0.006). Mean weekly PHQ-9 scores did not vary significantly across the study period (range: 2.53–3.21; *p* = 0.101), in contrast to mean FCV-19S scores which were highest during the early weeks of the survey but decreased significantly thereafter (range: 18.34–20.31; *p* <  0.001). The median Likert score for worrying about other people’s health was significantly higher than that of worrying for one’s own health: 1 (IQR: 1–3) vs 1 (IQR: 1–2), *p* <  0.001.

Using dichotomized PHQ-9 and FCV-19S scores, we estimated the prevalence of depression and high fear for the study weeks with ≥100 responses (Table 3). We observed similar proportions of respondents with depression across the different study weeks (*p* = 0.195), while there were significant differences in the prevalence of high fear (*p* <  0.001).

**Table 3:** Prevalence of depression and high fear across the study weeks

Calendar week	High fear (FCV-19S ≥ 19): n (%)	***P***-value^a^	Depression (PHQ-9 score ≥ 10): n (%)	***P***-value^a^
Week 30 (*n* = 817)	517 (63.3%)	< 0.001	74 (9.1%)	0.195
Week 31 (*n* = 286)	167 (58.4%)		27 (9.4%)	
Week 34 (*n* = 1132)	677 (59.8%)		84 (7.4%)	
Week 35 (*n* = 120)	68 (56.7%)		11 (9.2%)	
Week 36 (*n* = 640)	333 (52.0%)		32 (5.0%)	
Week 39 (*n* = 152)	90 (59.2%)		14 (9.2%)	
Week 40 (*n* = 454)	248 (54.6%)		36 (7.9%)	
Week 43 (*n* = 1686)	940 (55.8%)		159 (9.4%)	
Week 47 (*n* = 1347)	785 (58.3%)		132 (9.8%)	
Week 49 (*n* = 397)	216 (54.4%)		26 (6.6%)	

^a^Chi squared test

The multivariate analysis revealed that having a postgraduate degree, a history of quarantine, experiencing flu-like symptoms during the past 14 days, and higher FCV-19S scores were associated with increased odds to have more severe depressive symptoms. Meanwhile, obtaining COVID-19 information from either official sources or from the social media reduced these odds (Table 4).

**Table 4:** Clustered ordinal logistic regression investigating factors associated with increasing severity of depressive symptoms

Covariates	Univariate ***P***-value	Adjusted OR (95% CI)	Multivariate ***P***-value
Age (in years)	0.099	0.994 (0.986–1.003)	0.177
Socioeconomic status:			
Low	Ref	Ref	
Lower middle	0.097	1.137 (0.991–1.305)	0.066
Upper middle	0.809	1.041 (0.853–1.269)	0.694
High	0.117	1.300 (0.853–1.980)	0.222
Educational level			
Primary	Ref	Ref	
Secondary	0.337	1.354 (0.914–2.006)	0.131
Undergraduate	0.247	1.388 (0.919–2.097)	0.119
Postgraduate	0.141	1.523 (1.011–2.294)	0.044
Profession			
Student	Ref	Ref	
Jobless	0.767	1.026 (0.877–1.201)	0.746
Self-employed	0.173	0.974 (0.787–1.205)	0.808
Private worker	0.850	1.022 (0.928–1.127)	0.655
Government worker	0.614	0.953 (0.819–1.107)	0.526
Retired	0.914	1.210 (0.727–2.013)	0.463
Student / worker in healthcare sector	0.159	1.095 (0.958–1.252)	0.185
COVID-19 information from official sources (TV, radio, or other government channels)	0.011	0.801 (0.728–0.882)	< 0.001
COVID-19 information from social media	0.078	0.831 (0.758–0.912)	< 0.001
Living alone in household	0.132	1.098 (0.986–1.224)	0.089
Flu-like symptoms during the past 14 days	< 0.001	2.296 (2.050–2.570)	< 0.001
History of quarantine/isolation for COVID-19	< 0.001	1.568 (1.422–1.728)	< 0.001
History of violence/discrimination	0.182	0.949 (0.816–1.104)	0.501
Fear of COVID-19 score	< 0.001	1.114 (1.102–1.127)	< 0.001
Residential setting		–	–
Rural	Ref		
Sub-urban	0.639		
Urban	0.567		
COVID-19 information from healthcare workers	0.929	–	–
Male gender	0.459	–	–
Conflict-stricken region	0.329	–	–

*OR* Odd’s ratio, *CI* Confidence interval, *Ref* Reference category

## Discussion

Our study is the first to assess fear and depression among Cameroonian adults during 6 months of the COVID-19 crisis, in a nation-wide large survey. Overall, almost one-tenth of respondents screened positive for depression and the PHQ-9 depression scores appeared to vary proportionately to the weekly incidence of COVID-19. It is worth noting that during the latter months of the study period, schools were allowed to resume cautiously and this brought about a semblance of normalcy in the daily routines of Cameroonians. These changes may have had repercussions on the overall psychosocial well-being of the study participants over time.

The prevalence of depression during the COVID-19 crisis in our study is lower compared to previous reports by researchers who also used the PHQ-9 tool with a cut-off score of ≥10 in both high-income and low- middle-income countries [17, 20–22]. Our numbers are also lower than pre-COVID-19 depression rates in healthy Cameroonian adults, estimated at 19.8% in one study [23] but the fact that a different screening tool (Beck’s Depression Inventory) was used renders comparisons with our study difficult. It is expected that the COVID-19 pandemic would cause a rise in the burden of depression compared to baseline levels, as observed during a comparative study in American adults [24]. Of note, pre-COVID-19 depression levels were already low in Africa compared to other continents (except for Australia) [25], and the pandemic would likely exacerbate existing situations without necessarily altering the global distribution of depressive disorders. Furthermore, in resource-limited settings such as Cameroon where economic hardship is frequent, lower depression rates may also be observed because people are more focused on being financially productive amidst the crisis to keep providing for their families, even to the point of overlooking or mentally downplaying the threat of COVID-19. This notwithstanding, the most educated respondents (post-graduate level) who probably had a better understanding of the COVID-19 science and the risks associated with the pandemic were prone to more severe depressive symptoms (adjusted odds ratio of 1.5, relative to respondents with primary level education).

Regarding FCV-19S results, over half of the participants experienced high levels of fear vis-à-vis COVID-19. This is greater than the 35.7% reported in Greece [17] using a similar methodology. COVID-19 fear scores were highest at the beginning of the study period (June 2020), possibly because that period represented the first epidemic peak in Cameroon with thousands of cases confirmed on a weekly basis. No regional disparity was noted in the prevalence of depression or high fear of COVID-19. This is an intriguing finding, because we expected that persons residing in conflict-stricken regions would experience more psychological and social distress in the face of the COVID-19 outbreak. Qualitative research may be required to better understand the determinants of mental and brain health, as well as the coping mechanisms of individuals in different settings during the COVID-19 pandemic.

The frequency of depression and high fear of COVID-19 was similar among participants who were workers/students in the healthcare sector and others not involved in healthcare. A possible explanation for this observation is the reduced exposure to COVID-19 patients for health personnel in Cameroon due to a relatively lower COVID-19 burden compared to other regions of the world. Indeed, increased exposure to COVID-19 cases has been shown to fuel stress, anxiety and depression among healthcare providers [26]. Therefore, it is understandable that mental and brain disorders during the COVID-19 pandemic may be more frequent among healthcare workers in Europe, America, Asia [3, 6, 27–29], but also in neighbouring Nigeria [30] which has more than thrice the number of COVID-19 cases in Cameroon [1]. It is worth noting that another survey previously conducted among healthcare workers in Cameroon during the COVID-19 pandemic found a prevalence of depression of 42.8% using the Hospital Anxiety and Depression scale (HADS) [31], compared to 8.5% in our study. While acknowledging the methodological differences between our study and the aforementioned survey, their findings do suggest that exposure of healthcare workers to COVID-19 patients is an important stressor in Cameroon. The authors reported that fear of contamination with the coronavirus and fear of death were modulators of depression among the health personnel [31].

Participants with a history of quarantine, or those who had recently experienced flu-like symptoms (during the past 2 weeks) were more likely to exhibit depressive symptoms. These results concur with observations made in Canada during the SARS-1 outbreak during which longer quarantine durations and a history of contact with an infected person were associated with increased psychological distress [32]. Similar observations were made in Brazil and Portugal during the COVID-19 pandemic [33]. This highlights quarantine / isolation as a risk factor for short- and possibly long-term psychosocial distress, requiring careful consideration by the health authorities [34]. We observed that during the COVID-19 outbreak in Cameroon, the respondents were more concerned about the health of their loved ones than their own health. This finding is relevant in the context of implementing public health measures such as mandatory quarantine and vaccination for COVID-19. Although adherence to preventive measures may depend more on their practicality (ease of implementation) than on the psychology of individuals [35], one could still appeal to altruism by reminding the public about the community-wide benefits of such interventions. It is clear the impact of the interventions goes beyond individual protection to shielding other community members from becoming infected. In a situation whereby even healthcare workers are against a vaccine trial in their community [36], it is important to emphasize the collective benefits of vaccination while rendering the process of COVID-19 vaccination as simple and attractive as possible in a bid to improve acceptability. Studies on attitudes and acceptability of the COVID-19 vaccine in Cameroon are needed.

Participants who resorted to various platforms (radio, television, government announcements, or social media) in search of information about COVID-19 had reduced odds of experiencing depressive symptoms. Similar findings were reported by González-Sanguino et al. in Spain, as they found a negative relationship between depression and having adequate information about the coronavirus [9]. Meanwhile, a study conducted among Chinese adults during their nationwide lockdown showed that a higher level of COVID-19 media exposure was significantly associated with higher PHQ-9 scores [37]. We surmise that the information from various media rather produced a soothing effect on Cameroonian participants by unveiling how grave the COVID-19 situation was in other countries, and providing a sense of relative safety. Indeed, the COVID-19 burden and death toll in Cameroon is considerably lower compared to the daily reports from Europe and the Americas [38]. It is expedient that the media outlets be leveraged to promptly disseminate adequate COVID-19-related information to the public, as this approach is a pillar in ensuring optimal mental and brain health during such health crises by addressing any sense of uncertainty and fear [39].

A number of limitations must be taken into account when interpreting our study findings. This was an online survey, and it is therefore impossible to verify the veracity of the provided responses. In addition, we acknowledge a sampling bias since our survey could only be filled by literate and educated individuals who had access to internet. Indeed, the web-based recruitment procedures could not achieve a representative study population. Fewer female respondents participated in the survey; this could be due to the fact that in Cameroon, it was reported that women are less likely to use the internet compared to men [40]. After the preliminary data analysis, an effort was made through the Ministry of Women’s Empowerment and the Family to encourage more women in women’s groups to participate but the percentage increase in participation was just 1.5 (from 25.2 to 26.7). Since the female gender has been associated with greater psychological distress during the COVID-19 outbreak [9], the male predominance in our study population might have led to an underestimation of the overall burden of COVID-19-related fear and depression in Cameroon. Lastly, the survey’s duration was very long, considering the rapidly evolving dynamics of COVID-19 and could lead to participant fatigue. However, this was mitigated by constituting weekly clusters during the data analysis.

## Conclusions

Our study found that less than 10% of Cameroonian adults reported depressive symptoms amidst the COVID-19 crisis, much lower than what has been documented in other settings. Meanwhile, over half of the respondents experienced a high level of fear of COVID-19. We recommend that various media outlets should be leveraged to disseminate adequate information about COVID-19 as this will dispel fear and reduce the risk for depression. Furthermore, persons who report flu-like symptoms, the more educated individuals (postgraduates) and those who had been quarantined seem to be at increased risk for depression, and should be prioritized for interventions during the COVID-19 pandemic in Cameroon. Finally, appealing to the population’s sense of altruism may be the way forward to ensure optimal mental and brain health, as well as better acceptability of large scale COVID-19 preventive measures.

## Data Availability

All the data presented in this article are available upon reasonable request from the ICPcovid consortium: icpcovid@uantwerpen.be

## Abbreviations

COVID-19: Coronavirus disease 2019
FCV-19S: Fear of COVID-19 score
ICPCovid: International Citizen Project on COVID-19
IQR: Interquartile range
PHQ-9: Patient Health Questionnaire
